# A Dyadic Test of the Association Between Trait Self-Control and Romantic Relationship Satisfaction

**DOI:** 10.3389/fpsyg.2020.594476

**Published:** 2020-12-21

**Authors:** Pei-Ying Zuo, Johan C. Karremans, Anouk Scheres, Esther S. Kluwer, William J. Burk, Gesa Kappen, Hagar Ter Kuile

**Affiliations:** ^1^Behavioural Science Institute, Radboud University, Nijmegen, Netherlands; ^2^Department of Social, Health and Organizational Psychology, Utrecht University, Utrecht, Netherlands

**Keywords:** trait self-control, relationship satisfaction, relationship commitment, romantic relationships, dyadic, cross-sectional, longitudinal

## Abstract

Previous research has demonstrated that trait self-control is related to a range of positive romantic relationship processes, suggesting that trait self-control should be positively and robustly linked to relationship satisfaction in both partners in a romantic relationship. However, the existing empirical evidence is limited and mixed, especially regarding partner effects (i.e., the effect of one’s self-control on the partner’s relationship satisfaction). With three datasets of heterosexual couples (S1: *N* = 195 newlyweds, longitudinal; S2: *N* = 249 couples who transition into first parenthood, longitudinal; S3: *N* = 929 couples, cross-sectional), the present pre-registered studies examined: (1) the dyadic associations between trait self-control and relationship satisfaction both cross-sectionally and longitudinally, and (2) whether these effects hold when controlling for both partners’ relationship commitment. The results indicated a cross-sectional positive actor effect, some support for a positive cross-sectional partner effect, and only little support for a longitudinal actor (but not partner) effect. After controlling for relationship commitment, all effects of trait self-control on satisfaction diminished except for a longitudinal actor effect among women in Study 2. Potential explanations for the current results, and implications for theory and practice, are discussed.

## Introduction

Trait Self-control, defined as the ability to inhibit unwanted impulses and to respond in a goal-directed manner (Vohs and Baumeister, 2016, p. 2), is important in many life domains, including the functioning and wellbeing of romantic relationships (Finkel and Campbell, 2001; Karremans et al., 2015). Indeed, many studies found that trait self-control is associated with various relationship benefits, such as increased levels of perspective-taking (Tangney et al., 2004), responsiveness (Gomillion et al., 2014), constructive communication (Bornstein and Shaffer, 2017), sacrifice (Pronk and Karremans, 2014), forgiveness (Burnette et al., 2014), reductions in aggressiveness (Denson et al., 2012), and refraining from the temptation of attractive alternatives (Pronk et al., 2011).

Given these positive romantic relationship outcomes, it seems reasonable to assume that the higher one’s trait self-control, the higher romantic relationship satisfaction will be, in both the individual and the partner, and that the current level of trait self-control is predictive of future relationship satisfaction. There is evidence suggesting that couples are happier when there is more overall self-control in the relationship (Vohs et al., 2011). However, surprisingly, relatively few studies have focused explicitly on the association between trait self-control and relationship satisfaction while taking a dyadic and/or longitudinal approach, and as will be explained in more detail shortly, the existing support is somewhat mixed. Thus, it is not clear whether people high in trait self-control are actually more satisfied with their relationship, and importantly, whether their partners are also more satisfied with the relationship, both cross-sectionally and longitudinally. In the current research, we used two longitudinal couple datasets and a third large-scale cross-sectional dataset, to examine these associations. We examined whether consistent findings would emerge among samples with different relationship characteristics. In addition, we explored whether trait self-control plays a unique role in predicting relationship satisfaction when a core factor of relationship wellbeing, relationship commitment, is also considered.

### Trait Self-control and Relationship Satisfaction

How would trait self-control be associated with relationship satisfaction? It has been argued that self-control is a driving force directing gut-level destructive impulses towards constructive responses that are aligned with long-term relationship goals (Finkel and Campbell, 2001), a process called the transformation of motivation (Yovetich and Rusbult, 1994). Consistent with this reasoning, and as mentioned above, individuals with high trait self-control indeed are better able to exhibit pro-relationship behaviors towards the partner, especially when faced with dilemmas between responding on self-interested motives or partner- and relationship-oriented motives (e.g., constructive communication, forgiveness, sacrifice). Because they are more likely to do so, individuals with high self-control also tend to be perceived as more responsive (Gomillion et al., 2014) and trustworthy (Gomillion et al., 2014; Righetti and Finkenauer, 2011) by their partners. Based on such findings, one could predict that high trait self-control is associated with a high level of relationship satisfaction, both for oneself, and perhaps especially, for the partner.

However, the empirical support is mixed. There is some evidence for both positive cross-sectional and longitudinal *actor effects* of trait self-control on relationship satisfaction (i.e., is partner A’s level of trait self-control associated with partner A’s relationship satisfaction?), but only a little evidence for a positive cross-sectional (but not longitudinal) *partner effect* (i.e., is partner A’s level of trait self-control associated with partner B’s relationship satisfaction?). We found ten studies that employed a dyadic approach in examining the association between romantic relationship satisfaction and self-control (Vohs et al., 2011; Young, 2017), or related constructs that have large conceptual overlap with self-control, namely, constraint (Donnellan et al., 2007), impulsivity (Lavner et al., 2017), self-discipline versus impulsiveness (Patrick et al., 2007), and disinhibition (Watson et al., 2004). As for actor effects, the findings generally supported that own self-control indeed was positively associated with own concurrent relationship satisfaction (Donnellan et al., 2007; Lavner et al., 2017; Mead, 2005; Robins et al., 2000; Stroud et al., 2010; Tan et al., 2017; Watson et al., 2004; Young, 2017), and with own relationship satisfaction 9 months later (Vohs et al., 2011), but not with own relationship satisfaction later on (e.g., 4 years’ trajectories of marital satisfaction; Lavner et al., 2017). As for partner effects, only a few studies found that own self-control was positively associated with the partner’s concurrent relationship satisfaction (Mead, 2005; Patrick et al., 2007; Tan et al., 2017). However, one study found no actor nor partner effects (Stroud et al., 2010). Moderating effects of gender (Robins et al., 2000) and relationship status (e.g., dating versus married couples, the number of children; Stroud et al., 2010), and varying results with different measures (Robins et al., 2000; Stroud et al., 2010), make the findings even more ambiguous.

What may explain these mixed findings? Although there may be various reasons (we return to this issue more extensively in the General Discussion), one plausible reason may be that the effects of self-control are relatively small as compared to the effects of broader relationship motives, specifically, relationship commitment. Relationship commitment is defined as the motivation to stay in a relationship and having a long-term orientation (Rusbult and Buunk, 1993), and is rooted in past relationship experiences. Rooted in interdependence theory (Rusbult and Van Lange, 2003), relationship commitment can be considered as a major relationship-specific motive (i.e., macro-motive; Holmes and Rempel, 1989) that plays a central role in the functioning and wellbeing of romantic relationships. Existing literature has documented that relationship commitment is associated with positive feelings and thoughts about the partner and the relationship (Rusbult and Buunk, 1993), trust (Wieselquist et al., 1999), forgiveness (Finkel et al., 2002), intimacy (Acker and Davis, 1992), and a range of other beneficial relationship outcomes (Stanley et al., 2010). Furthermore, a large body of research has shown that commitment (Givertz et al., 2016; Hendrick et al., 1988) is strongly associated with relationship satisfaction. It is important to note that relationship satisfaction can both be a determinant as well as an outcome of relationship commitment, affecting each other in a cyclical manner (e.g., satisfaction promotes commitment, and commitment promotes relationship satisfaction by promoting pro-relationship responses; Wieselquist et al., 1999). Considering the importance of commitment for relationship satisfaction, an interesting and important question that we aim to answer is whether the effects of trait self-control on relationship satisfaction occur *above and beyond* the effects of relationship commitment. Or put differently, when the commitment, the motivation to stay in a relationship, is strong, does self-control play any additional role in promoting relationship satisfaction? This question also speaks to the broader issue of whether relationship satisfaction is determined mainly by relationship-specific factors, or is determined mainly or additionally by individual difference factors of both partners (Joel et al., 2020).

### The Current Research

The goal of the current research was to test whether trait self-control has a robust and replicable association with own and the partner’s relationship satisfaction, concurrently and longitudinally, and whether any such associations would hold when a broader macro-motive (i.e., relationship commitment) is taken into account. We explored the actor and partner effects across the three datasets (see pre-registration, https://osf.io/hc5gt/?view_only=753af6222c7545dd9df5991b353dac9b). In all three studies, self-control was operationalized in terms of participants’ self-reported level of self-control^1^. We first re-analyzed a longitudinal dataset (Study 1, 195 heterosexual newlyweds, 5 waves’ annual evaluation) to test both the cross-sectional and longitudinal actor and partner effects of trait self-control on relationship satisfaction. Second, we analyzed another longitudinal dataset (Study 2, 249 heterosexual couples who went through the transition to parenthood) to examine whether the results of Study 1 could be replicated. Third, we used a large cross-sectional couple study (Study 3, 929 heterosexual couples whose relationship lengths ranged from about 8 to 37 years), with greater precision and power to obtain reliable estimates of concurrent associations between trait self-control and relationship satisfaction^2^.

## Study 1

### Materials and Methods

#### Participants

The original sample consisted of 199 heterosexual newlywed couples (five waves with annual assessments; for a description of the first two waves of the study, see Finkenauer et al., 2009; for a description of the waves 3, 4, and 5 of the study, see Muusses et al., 2015) in the Netherlands. Men and women in the first assessment were 32.91 (*SD* = 4.87) and 29.97 (*SD* = 4.25) years old, respectively. Relationship length was 5.71 years on average (*SD* = 3.03). For the current study, we made an *a priori* decision to use only self-report data of wave 2 (Time 1, 195 couples) and wave 5 (Time 2, 141 couples), as only wave 2 included all predictors of interest. At Time 1 (T1), 37.44% of the couples had children, while 94.33% had children at Time 2 (T2). Independent samples *t*-tests indicated that couples who dropped out at T2 did not differ from those who completed the T2 assessment on relationship commitment and relationship satisfaction (*p’*s ≥ .332), but they did differ on trait self-control. Men who dropped out scored higher on trait self-control at T1 than those who did not [*t*(193) = -1.98, *p* = 0.049], while women who dropped out were lower in trait self-control than those who did not [*t*(193) = 0.71, *p* = 0.027].

#### Measures

All the measures were in Dutch.

#### Trait Self-Control

The 11-item version of the Brief Self-Control Scale (BSCS; used in Finkenauer et al., 2005; Tangney et al., 2004) was used to assess trait self-control at T1. The original scale shows adequate reliability (Tangney et al., 2004) and structural validity (Manapat et al., 2019). The short Dutch version of the scale showed adequate reliability (Finkenauer et al., 2005; Frijns et al., 2005). Example items were “I have a hard time breaking bad habits,” and “I am good at resisting temptation.” Items were rated on a 5-point scale (1 = *not at all like me* to 5 = *very much like me*). Higher average scores indicated higher levels of trait self-control. Cronbach’s alphas for men and women were 0.74 and 0.71, respectively.

#### Relationship Commitment

An 8-item commitment scale (revised from the Investment Model Scale; Rusbult et al., 1998) was used at T1. The original scale shows good reliability, as well as convergent, discriminant, and predictive validity (Rusbult et al., 1998). Example items were “I want our relationship to last for a very long time,” and “I would not feel very upset if our relationship were to end in the near future” (reversed; 1 = *not true at all* to 5 = *completely true*). Higher average scores indicated higher levels of relationship commitment. Cronbach’s alphas for men and women were 0.90 and 0.91, respectively.

#### Relationship Satisfaction

The 10-item Dyadic Satisfaction Subscale of the Dyadic Adjustment Scale (Spanier, 1976) was used at T1 and T2. The original scale shows high reliability, as well as content, criterion-related, and construct validity (Spanier, 1976). Sample items are “How happy are you and your husband/wife - all in all - with your marriage?” (1 = *extremely unhappy* to 7 = *perfect*) and “How often do you think things are going well between you and your husband/wife? (1 = *never* to 6 = *always*).” Higher average scores indicated greater relationship satisfaction. Cronbach’s alphas for men and women at T1 and T2 ranged from 0.68 to 0.79.

#### Statistical Analysis

Descriptive analyses were carried out in SPSS 25.0. To test the main hypotheses, we used the actor–partner interdependence model (APIM; Kenny et al., 2006, p. 145) with structural equation modeling using the Lavaan package (Rosseel, 2012) in R Core Team (2013). In the current study, all variables were mixed variables (i.e., variables that could differ both across and within couples; Kenny et al., 2006, p. 9). Given the existing evidence for gender differences on the association between trait self-control and relationship satisfaction (Robins et al., 2000), we considered the heterosexual couples as distinguishable dyads in all the dyadic analyses in the current research. First, to investigate the cross-sectional effects of trait self-control on relationship satisfaction, we ran a basic APIM with both partners’ trait self-control at T1 predicting both partners’ relationship satisfaction at T1. Second, we ran another APIM controlling for both partners’ relationship commitment at T1. Third, we investigated the longitudinal effects of trait self-control on relationship satisfaction with another basic APIM, in which both partners’ trait self-control at T1 predicted both partners’ relationship satisfaction at T2, while controlling for both partners’ relationship satisfaction at T1. Fourth, we ran another APIM in which both partners’ relationship commitment at T1 were added to the model. To adjust for univariate and multivariate non-normality, all the models applied maximum likelihood estimation with robust (Huber-White) standard errors and a scaled test statistic that is (asymptotically) equal to the Yuan-Bentler test statistic, for both complete and incomplete data. We used full information maximum likelihood (fiml) to handle the missing data. Since the four APIMs were saturated models, which estimate *p*^∗^ parameters and fit the data perfectly (West et al., 2012), we used the sampling-error-adjusted Bayesian information criterion (SABIC) as the fit index (Garcia et al., 2015). All the models were tested among samples with and without outliers, which generated similar findings. Thus, for the final models, we used all the available data (i.e., with outliers). The same data analysis strategy was used for all three studies (i.e., cross-sectional effects in Studies 1, 2 and 3; longitudinal effects in Studies 1 and 2).

### Results

#### Descriptive Statistics

Descriptive statistics are presented in Table 1. The Pearson correlational analysis (two-tailed) provided support for a positive actor effect of trait self-control on relationship satisfaction, both cross-sectionally and longitudinally, but no support for partner effects, except that men’s trait self-control was associated with women’s concurrent relationship satisfaction.

**TABLE 1:** Correlations between variables in Study 1 (*N* = 195).

	***N***	***Mean* (*SD*)**	**1**	**2**	**3**	**4**	**5**	**6**	**7**	**8**
**Men**										
1. T1 Trait self-control	195	3.30 (0.47)								
2. T1 Relationship commitment	195	4.59 (0.45)	0.15*							
3. T1 Relationship satisfaction	195	4.27 (0.38)	0.36***	0.59***						
4. T2 Relationship satisfaction	138	4.16 (0.42)	0.31***	0.46***	0.68***					
**Women**										
5. T1 Trait self-control	195	3.18 (0.43)	-0.09	0.08	0.06	-0.06				
6. T1 Relationship commitment	194	4.65 (0.41)	0.02	0.13	0.18*	0.11	0.12			
7. T1 Relationship satisfaction	195	4.16 (0.43)	0.15*	0.31***	0.36***	0.24**	0.14^#^	0.42***		
8. T2 Relationship satisfaction	141	4.10 (0.42)	0.11	0.30***	0.36***	0.41***	0.17*	0.31***	0.52***	

*p < 0.05, **p < 0.01, ***p < 0.001, *^#^p* = 0.050, Two-tailed.

#### Cross-Sectional Actor and Partner Effects of Trait Self-Control on Relationship Satisfaction

**FIGURE 1:**
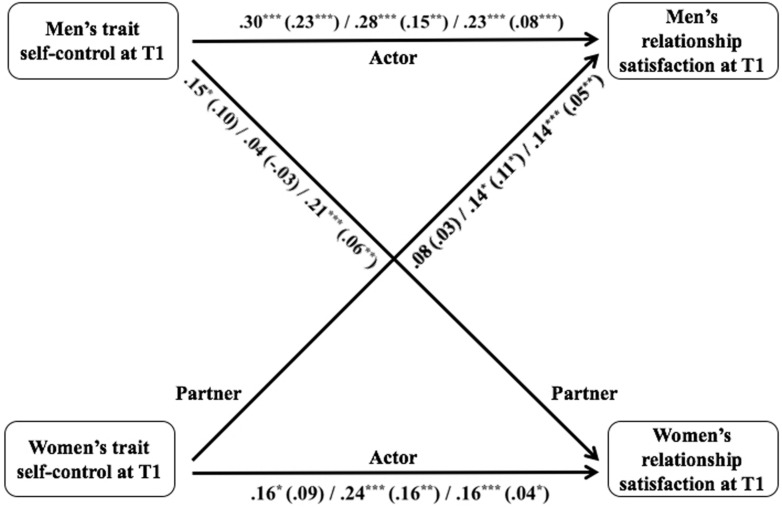
Cross-sectional Actor and Partner Effects of Trait Self-control on Relationship Satisfaction across Studies. Statistics illustrate the effects (unstandardized regression coefficients) in Studies 1, 2 and 3 respectively. Statistics in parentheses illustrate the effects when controlling for relationship commitment at T1. ∗*p* < 0.05. ∗∗*p* < 0.01. ∗∗∗*p* < 0.001.

Cross-sectional APIM statistics are summarized in Table 4 and Figure 1 (for detailed statistics of each model, see Table A1 in Supplementary Material A). Consistent with the correlational analysis, the results indicated positive actor effects of trait self-control on relationship satisfaction for men (*b* = 0.30, *SE* = 0.06, *p* < 0.001) and women (*b* = 0.16, *SE* = 0.06, *p* = 0.010). In addition, the results indicated a significant partner effect of trait self-control on relationship satisfaction for women (*b* = 0.15, *SE* = 0.08, *p* = 0.047), but not for men (*b* = 0.08, *SE* = 0.06, *p* = 0.165). That is, men’s levels of trait self-control were positively associated with their female partner’s current relationship satisfaction, whereas women’s levels of trait self-control were not significantly associated with their male partner’s current relationship satisfaction.

**TABLE 2:** Correlations between variables in Study 2 (*N* = 249).

	***N***	***Mean* (*SD*)**	**1**	**2**	**3**	**4**	**5**	**6**	**7**	**8**
**Men**										
1. T1 Trait self-control	236	3.23 (0.55)								
2. T1 Relationship commitment	233	4.82 (0.32)	0.23***							
3. T1 Relationship satisfaction	233	4.49 (0.52)	0.29***	0.62***						
4. T2 Relationship satisfaction	119	4.41 (0.65)	0.20*	0.37***	0.59***					
**Women**										
5. T1 Trait self-control	247	3.15 (0.52)	0.03	0.05	0.15*	0.20*				
6. T1 Relationship commitment	247	4.89 (0.22)	0.07	0.15*	0.18**	0.27**	0.14*			
7. T1 Relationship satisfaction	247	4.52 (0.51)	0.06	0.28***	0.39***	0.46***	0.24***	0.54***		
8. T2 Relationship satisfaction	136	4.53 (0.55)	0.02	0.26**	0.24**	0.60***	0.31***	0.31***	0.53***	

*p < 0.05, **p < 0.01, ***p < 0.001, Two-tailed.

**TABLE 3:** Correlations between variables in Study 3 (*N* = 929).

	***N***	***Mean* (*SD*)**	**1**	**2**	**3**	**4**	**5**	**6**
**Men**								
1. Trait self-control	929	4.85 (1.24)						
2. Relationship commitment	929	6.31 (0.91)	0.29***					
3. Relationship satisfaction	929	5.83 (0.96)	0.33***	0.73***				
**Women**								
4. Trait self-control	929	4.88 (1.21)	0.17***	0.17***	0.22***			
5. Relationship commitment	929	6.32 (0.94)	0.24***	0.51***	0.54***	0.22***		
6. Relationship satisfaction	929	5.74 (1.04)	0.28***	0.51***	0.66***	0.23***	0.76***	

*p < 0.05, **p < 0.01, ***p < 0.001, Two-tailed.

**TABLE 4:** Statistic summary on the actor and partner effects of trait self-control on relationship satisfaction across the three studies.

	**The basic APIM models**		**The APIM models controlling for relationship commitment**	
	**Men**	**Women**	**Men**	**Women**
**Cross-sectional models**				
**Actor effect^*a*^**				
Study 1 (*N* = 195)	0.30***	0.16*	0.23***	0.09
Study 2 (*N* = 249)	0.28***	0.24***	0.15**	0.16**
Study 3 (*N* = 929)	0.23***	0.16***	0.08***	0.04^∗^
**Partner effect^*b*^**				
Study 1	0.08	0.15*	0.03	0.10
Study 2	0.14*	0.04	0.11*	-0.03
Study 3	0.14***	0.21***	0.05**	0.06**
**Longitudinal models**^*c*^				
**Actor effect**				
Study 1	0.04	0.11	0.04	0.11
Study 2	0.05	0.19*	0.05	0.21*
**Partner effect**				
Study 1	-0.06	-0.05	-0.06	-0.06
Study 2	0.04	0.00	0.06	0.00

^*a*^Actor effect indicates the effect of partner A’s predictor on partner A’s relationship satisfaction (unstandardized regression co-efficients). ^*b*^Partner effect indicates the effect of partner B’s predictor on partner A’s relationship satisfaction (unstandardized regression co-efficients). ^*c*^Effects in the longitudinal models illustrate the effects of trait self-control on the change of relationship satisfaction between T1 and T2 (i.e., the slope). *p < 0.05, **p < 0.01, ***p < 0.001.

When adding partners’ relationship commitment to the model, the association between men’s own trait self-control and own concurrent relationship satisfaction remained significant (*b* = 0.23, *SE* = 0.04, *p* < 0.001), but the actor effect for women disappeared (*b* = 0.09, *SE* = 0.06, *p* = 0.141), and there were no significant partner effects for both genders (men: *b* = 0.03, *SE* = 0.05; women: *b* = 0.10, *SE* = 0.06; *p’s* ≥ 0.084). Additionally, relationship commitment was a significant predictor of concurrent relationship satisfaction, as an actor effect for both genders (men, *b* = 0.46, *SE* = 0.08; women, *b* = 0.44, *SE* = 0.12; *p’*s < 0.001), and a partner effect for women (women, *b* = 0.21, *SE* = 0.07; *p* = 0.002), but not for men (*b* = 0.07, *SE* = 0.05, *p* = 0.203).

#### Longitudinal Actor and Partner Effects of Trait Self-Control on Relationship Satisfaction

**FIGURE 2:**
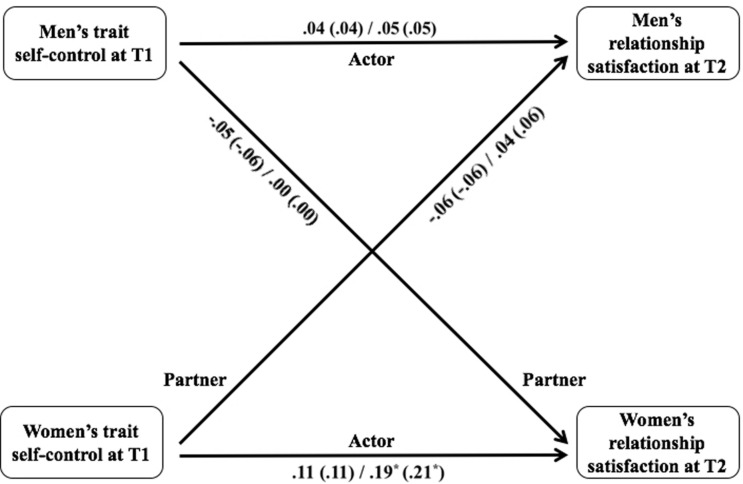
Longitudinal Actor and Partner Effects of Trait Self-control on Relationship Satisfaction in Studies 1 and 2. Statistics illustrate the effects (unstandardized regression coefficients) of trait self-control on the change of relationship satisfaction between T1 and T2 (i.e., the slope) in Studies 1 and 2 respectively. Statistics in parentheses illustrate the effects when controlling for relationship commitment and relationship satisfaction at T1. ∗*p* < 0.05. ∗∗*p* < 0.01. ∗∗∗*p* < 0.001.

Longitudinal APIM statistics are presented in Table 4 and Figure 2 (for detailed statistics of each model, see Table A2 in Supplementary Material A). The results showed no significant actor (men, *b* = 0.04; women, *b* = 0.11; *SE’*s = 0.07, *p’*s ≥ 0.136) or partner effects (men, *b* = -0.06, *SE* = 0.07; women, *b* = -0.05, *SE* = 0.06, *p’*s ≥ 0.362) of trait self-control on relationship satisfaction 3 years later for both genders. Controlling for both partner’s relationship commitment did not change the significance of these results (*p’*s ≥ 0.139). Thus, for both genders, trait self-control did not predict one’s own or the partner’s relationship satisfaction 3 years later. Additionally, there was no actor (men, *b* = 0.05, *SE* = 0.08; women, *b* = -0.01, *SE* = 0.09; *p’*s ≥ 0.563) or partner effects (men, *b* = -0.05, *SE* = 0.06; women, *b* = 0.01, *SE* = 0.10; *p’*s ≥ 0.445) of relationship commitment on satisfaction 3 years later.

### Discussion

In sum, Study 1 found some support for both positive actor and partner effects of trait self-control on relationship satisfaction. However, these effects only emerged cross-sectionally, with no evidence for any longitudinal effect. We found some gender differences, such that cross-sectionally men’s levels of trait self-control were associated with their female partner’s relationship satisfaction, but women’s levels of trait self-control were not associated with their male partner’s relationship satisfaction. Importantly, when both partners’ relationship commitment was taken into account, both cross-sectional actor and partner effects diminished.

## Study 2

We used another existing couple data set (Study 2) to test the replicability and robustness of both the cross-sectional and longitudinal effects we found in Study 1.

### Materials and Methods

#### Participants

The original dataset consisted of 440 Dutch men and women who were going through the transition to parenthood (Ter Kuile et al., in press). Participants either received 20 euros upon completion of the fourth wave’s assessment or participated in a lottery for one prize of 250 euro and five prizes of 50 euros. With online questionnaires, four waves of data were collected during pregnancy, and when the child was approximately four months old, eight months old, and one year old (about 1.5 years after the first assessment). To make the current study comparable with Study 1, we used only two waves’ data (T1: wave 1, 249 couples; T2: wave 4, 139 couples) and included only heterosexual couples of which both partners’ data were available in the first wave. Independent samples ***t***-tests indicated that couples who dropped out at T2 did not differ from those who completed the T2 assessment on the key variables of the current study (*p*’s > 0.095). At T1, the mean ages of men and women were 30.72 (***SD*** = 4.72) and 28.06 (***SD*** = 3.72) years old, respectively. The average relationship length was 6.25 years (***SD*** = 3.53). Half of the couples (52%) were married, 29% were living together, and 19% were cohabiting with a cohabitation contract. Most respondents received their highest education in an applied/scientific university (60.2% for men, 77% for women), while around one-third of them completed primary/high school or basic vocational education (37.8% for men, 31% for women). Around half of them had a monthly income of less than 2000 euros (men, 48.5%; women, 69.2%), and the rest mainly had an income between 2000 to 3000 euros (men, 41.6%; women, 28%).

#### Measures

All the measures were in Dutch.

#### Trait Self-Control

The 11-item version BSCS (used in Finkenauer et al., 2005; Tangney et al., 2004) was used at T1 as in Study 1. Cronbach’s alphas for men and women were 0.75 and 0.76, respectively.

#### Relationship Commitment

In this study, a 5-item^3^ commitment scale (Arriaga and Agnew, 2001) was used at T1. Sample items were “I intend to stay in this relationship” and “I feel strongly attached to our relationship” (1 = *totally disagree* to 5 = *totally disagree*). Higher average scores indicated higher levels of relationship commitment. Cronbach’s alphas for men and women were low, 0.68 and 0.59, respectively.

#### Relationship Satisfaction

A 5-item Satisfaction Subscale of the Investment Model Scale (Rusbult et al., 1998) was used at T1 and T2. The original scale shows good reliability, as well as convergent and discriminant validity (Rodrigues and Lopes, 2013; Rusbult et al., 1998). Sample items were “Our relationship makes me very happy” and “My relationship is much better than others’ relationships” (1 = *completely disagree* to 5 = *completely agree*). Higher average scores indicated greater relationship satisfaction. Cronbach’s alphas for men and women at T1 and T2 ranged from 0.81 to 0.89.

### Results

#### Descriptive Statistics

Descriptive statistics are presented in Table 2. Consistent with Study 1, the correlation analysis (two-tailed) provided support for the cross-sectional and longitudinal actor effects of trait self-control on relationship satisfaction for both genders, and non-significant longitudinal partner effect on women’s relationship satisfaction. Other than in Study 1, significant partner effects on men’s satisfaction were found, both cross-sectionally and longitudinally. Additionally, in contrast to Study 1, there was no cross-sectional partner effect on women’s relationship satisfaction.

#### Cross-Sectional Actor and Partner Effects of Trait Self-Control on Relationship Satisfaction

As shown in Table 4 and Figure 1 (for detailed statistics for each model, see Table A3 in Supplementary Material A), the results of the APIM indicated a positive actor effect of trait self-control on relationship satisfaction for both men (*b* = 0.28, *SE* = 0.07, *p* < 0.001) and women (*b* = 0.24, *SE* = 0.06, *p* < 0.001), which is consistent with Study 1. Different from Study 1, we found a positive partner effect on men’s satisfaction (*b* = 0.14, *SE* = 0.07, *p* = 0.038), but not on women’s satisfaction (*b* = 0.04, *SE* = 0.06, *p* = 0.470). When controlling for both partners’ commitment, even though the effect sizes diminished, the significance levels of all effects on satisfaction did not change for both genders (actor effect: men, *b* = 0.15; women, *b* = 0.16; *SE’*s = 0.05, *p’*s = 0.001; partner effect: men, *b* = 0.11, *SE* = 0.05, *p* = 0.044; women, *b* = −0.03, *SE* = 0.04, *p* = 0.432). These findings are consistent with Study 1, with the exception that women’s trait self-control was still significantly associated with their own and their male partner’s concurrent relationships satisfaction after controlling for commitment.

Additionally, and similar to the findings in Study 1, there was a significant actor effect of commitment on concurrent relationship satisfaction for both genders (men, *b* = 0.92, *SE* = 0.12; women, *b* = 1.16, *SE* = 0.18; *p’*s < 0.001), and a partner effect for women (*b* = 0.33, *SE* = 0.14, *p* = 0.021), but not for men (*b* = 0.16, *SE* = 0.15, *p* = 0.280).

#### Longitudinal Actor and Partner Effects of Trait Self-Control on Relationship Satisfaction

Longitudinal APIM statistics are presented in Table 4 and Figure 2 (for detailed statistics for each model, see Table A4 in Supplementary Material A). Consistent with Study 1, the results indicated that there was no longitudinal actor effect on men’s satisfaction (*b’*s = 0.05; *SE’*s = 0.10; *p’*s ≥ 0.582), regardless of whether or not controlling for both partners’ commitment. However, different from Study 1, the data of Study 2 yielded a positive longitudinal actor effect of trait self-control at T1 on relationship satisfaction 1.5 years later among women (*b* = 0.19, *SE* = 0.08, *p* = 0.021), even when controlling for commitment (*b* = 0.21, *SE* = 0.08, *p* = 0.01). There were no partner effects on men’s (*b’*s = 0.04 and 0.06; *SE’*s = 0.12; *p’*s ≥ 0.637) nor women’s satisfaction (*b’*s = 0.00; *SE’*s = 0.09 and 0.08; *p’*s ≥ 0.966), which is consistent with Study 1. Additionally, no longitudinal actor nor partner effects of commitment on relationship satisfaction were found for either genders (actor effect: men, *b* = 0.17, *SE* = 0.22; women, *b* = 0.20; *SE* = 0.34; partner effect: men, *b* = 0.32, *SE* = 0.38; women, *b* = 0.29, *SE* = 0.17, *p’*s ≥ 0.093).

### Discussion

In replication of Study 1, Study 2 obtained positive cross-sectional actor effects of trait self-control for both genders, and some support for a partner effect. In contrast to Study 1, the partner effect now occurred for men (and not for women, as in Study 1), meaning that women’s levels of trait self-control were associated with their male partner’s relationship satisfaction. Different from Study 1, the cross-sectional actor effect still emerged for women when taking commitment into account, as well as a partner effect for men. Other than in Study 1, we also found support for a longitudinal actor effect for women, even when controlling for commitment.

## Study 3

Study 3 was a large-scale study that we used to provide a well-powered validation for the cross-sectional effects that were found in Studies 1 and 2.

### Materials and Methods

#### Participants

We used data from a study among 1233 romantic couples who were invited to participate in a two-week couple intervention (Karremans et al., 2020). Before the intervention, all participating couples were asked to fill in questionnaires that included trait self-control, relationship commitment and relationship satisfaction. We used these baseline data for the current study. Participants who were currently involved in a romantic relationship with a minimum duration of one year, living together with their partner, and 18 years or older were recruited via an independent Dutch research agency^4^, which has a nation-wide participant panel. Qualified participants were invited to fill in the informed consent, and completed the questionnaires. In the current study, we included data from heterosexual couples of which both partners completed the questionnaires (*N* = 929 couples). Men and women were on average 50.59 (*SD* = 14.10) and 47.77 (*SD* = 13.76) years old, respectively. Relationship length was 22.45 years on average (*SD* = 14.57). Most couples (92.9%) were living together, 71.8% were married and 73.6% had at least one child. Nearly half of the participants received their highest education from an applied/scientific university (45.4% of the men, and 43.5% of the women), while the rest had a high school, vocational education or less. The gross annual salary of all household members was almost evenly distributed: 18.7% were below 34,500 euros, 21.5% were between 34,500 euros and 41,200 euros, 26.2% were between 41,200 euros and 69,000 euros, 15.5% were equal to or beyond 69,000 euros.

#### Measures

All the measures were in Dutch.

#### Trait Self-control

We used the 4-item self-restraint subscale of Barkley deficits in executive functioning scale (adults and short version; Barkley, 2011, p. 154). The original scale shows good reliability (Barkley, 2011, p. 71), as well as construct and criterion validity (Barkley, 2011, p. 73). Participants rated to what extent the items described their behavior during the past 6 month (1 = never or rarely to 7 = very often). Example items were “unable to inhibit my reactions or response toward events or others (reversed),” and “acting without thinking (reversed).” Higher average scores indicated higher levels of self-control. Cronbach’s alphas for men and women were 0.84 and 0.83, respectively. While this measure is different from the self-control measures used in Studies 1 and 2, there is a large conceptual overlap in the measures, and in our previous research (Zuo et al., 2018), the correlations between Tangney’s scale (i.e., the 13-item version BSCS) and Barkley’s measure were 0.57 for men and 0.48 for women, respectively (one-tailed, *p*’s < 0.001).

#### Relationship Commitment

The 7-item commitment subscale of the Investment Model Scale (Rusbult et al., 1998) was used, as in Study 1. Cronbach’s alphas for men and women were 0.84 and 0.83, respectively.

#### Relationship Satisfaction

A 7-item Relationship Assessment Scale (Hendrick, 1988) was used. The original scale shows good reliability and construct validity (Hendrick, 1988). Sample items are “How satisfied are you with your relationship,” and “How many problems are there in your relationship (reversed)” (1 = *low satisfaction* to 5 = *high satisfaction*). Higher average scores indicated higher levels of relationship satisfaction. Cronbach’s alpha was 0.92 for men and 0.93 for women.

### Results

#### Descriptive Statistics

Descriptive statistics are presented in Table 3. The correlation analysis (two-tailed) provided consistent support for the cross-sectional actor effects of trait self-control on relationship satisfaction for both genders. Unlike Studies 1 and 2, cross-sectional partner effects were significant for both genders.

#### Cross-Sectional Actor and Partner Effects of Trait Self-Control on Relationship Satisfaction

Cross-sectional APIM statistics are summarized in Table 4 and Figure 1. Detailed statistics for each model are presented in Table A5 (see Supplementary Material A). Consistent with the correlation analysis, the results indicated a positive actor effect of trait self-control on relationship satisfaction for both genders (men: *b* = 0.23, *SE* = 0.02; women: *b* = 0.16, *SE* = 0.03; *p’*s < 0.001), which is consistent with the findings in Studies 1 and 2. A significant partner effect also emerged for both genders (men: *b* = 0.14; women: *b* = 0.21; *SE’*s = 0.03; *p’*s < 0.001). After controlling for both partners’ commitment, the significance of all effects did not change, but the effect sizes diminished (actor effect: men, *b* = 0.08, *SE* = 0.02, *p* < 0.001; women, *b* = 0.04, *SE* = 0.02, *p* = 0.028; partner effect: men, *b* = 0.05, *SE* = 0.02, *p* = 0.006; women, *b* = 0.06, *SE* = 0.02, *p* = 0.001). Thus, in Study 3, positive actor and partner effects of trait self-control on concurrent relationship satisfaction were found for both sexes, but the associations were weaker after controlling for relationship commitment.

Similar to the findings in Studies 1 and 2, both partners’ own commitment were significant predictors of their own concurrent relationship satisfaction (i.e., actor effect; men, *b* = 0.62, *SE* = 0.04; women, *b* = 0.73; *SE* = 0.03; *p’s* < 0.001), and men’s levels of commitment significantly predicted their female partner’s concurrent relationship satisfaction (i.e., a partner effect for women, *b* = 0.16; *SE* = 0.03; *p’*s < 0.001). However, different from Studies 1 and 2, women’s levels of commitment now were significantly associated with their male partner’s concurrent relationship satisfaction (i.e., a partner effect for men, *b* = 0.21, *SE* = 0.04, *p* < 0.001).

### Discussion

Thus, the findings of Study 3 provided cross-sectional support for both actor and partner effects regarding the association between trait self-control and relationship satisfaction, and again, the effects diminished when controlling for relationship commitment. We did not find gender differences that were consistent with the gender differences obtained in Study 1 or Study 2.

## General Discussion

In three studies, actor-partner interdependence models yielded some support for the prediction that both men and women were currently more satisfied with their relationship to the extent that they reported higher levels of trait self-control. This actor effect remained significant after controlling for relationship commitment in Studies 2 and 3 (except for women in Study 1). Importantly, the data showed little consistent support for partner effects, especially in Studies 1 and 2. These studies also showed some gender differences, but not consistent across studies. However, in the high-powered Study 3, both men and women were currently more satisfied with their relationship when their romantic partner reported higher levels of trait self-control, even when commitment was considered. Longitudinally, we found a positive actor (but not partner) effect among women in Study 2 only, independent of commitment. There were no other longitudinal partner effects for trait self-control. Across the three studies, we found a consistent positive actor effect of relationship commitment on concurrent relationship satisfaction for both genders, and a consistent positive partner effect of relationship commitment on concurrent relationship satisfaction for women (but not for men). In sum, the present findings suggest that trait self-control has a positive association with one’s own relationship satisfaction that is small to medium in magnitude, a less robust association with the partner’s relationship satisfaction, and all associations diminished when considering the role of relationship commitment, except for a longitudinal actor effect among pregnant women in Study 2.

In light of the large literature on the role of self-control in promoting relationship-beneficial processes, the current findings may seem surprising at first sight. Self-control has been associated with a variety of pro-relationship responses (e.g., forgiveness, sacrifice, and resisting tempting alternatives) that can be expected to contribute to both one’s own *and* the partner’s relationship satisfaction. However, trait self-control had only a relatively small impact on relationship satisfaction, particularly concurrently, as compared to the effects of a more motivational construct as commitment. Empirically, the effect sizes in the correlational findings were about twice as large for commitment as for trait self-control, and trait self-control explained less variance in concurrent relationship satisfaction than commitment. Neither trait self-control nor commitment effectively predicted relationship satisfaction longitudinally, with one exception in Study 2 (i.e., pregnant women’s trait self-control, but not commitment, predicted their own satisfaction 1.5 years later, even though the effects of trait self-control and commitment were similar in magnitude).

Although we do not want to suggest that ability factors like trait self-control do not play any role in determining relationship satisfaction, the current findings do suggest that when motivated – being highly committed to the relationship – partners may come a long way in maintaining a relatively satisfying relationship, irrespective of one’s own or the partner’s level of trait self-control. Interestingly, the current findings echo the results of a recent large-scale study (using machine learning), showing that relationship satisfaction is mainly explained by relationship-specific variables (like commitment), and that a range of individual difference variables does not add much predictive power in explaining relationship satisfaction or quality (Joel et al., 2020). One explanation may be that relationship-specific variables by definition were measured in the context of the relationship, whereas individual difference variables, like trait self-control, were not. Perhaps a different picture may emerge if self-control would have been measured regarding the specific context of the relationship (i.e., to what extent one exerts self-control ability in the context of his/her romantic relationship; see Slatcher and Vazire, 2009). Moreover, perhaps individual differences may exert a relatively distal and indirect effect on relationship satisfaction. The fact that the association between self-control and satisfaction diminished when controlling for commitment, may reflect such an indirect model: self-control may promote pro-relationship responses (as shown in previous research), resulting in stronger relationship commitment in both self and the partner through a dyadic process, which ultimately results in higher relationship quality and satisfaction.

Another possible and theoretically interesting reason for the relatively weak association between trait self-control and relationship satisfaction is that opposing forces may be at work. That is, whereas high self-control generally leads to positive relationship outcomes as previous research has indicated, there may be ‘hidden’ relationship costs to high self-control, and ‘hidden’ benefits of low self-control, that have received little theoretical and empirical attention so far. Koval et al. (2015) found that partners with high self-control experienced a greater burden from the partner relying on them, which could undermine their relationship satisfaction. Moreover, individuals with *low* self-control are viewed as more spontaneous and interesting (Zabelina et al., 2007), are less predictable (van Steenbergen et al., 2014), and display more non-normative behaviors (DeBono et al., 2011), making the relationship potentially more exciting and therefore satisfying (Reissman et al., 1993). Such processes may partly compensate for the general positive relationship outcomes of high self-control. Thus, the link between self-control and relationship satisfaction is possibly less straightforward than often assumed. More research is required to further explain the current findings, and explore the potential benefits of low self-control and costs of high self-control may be a fruitful direction.

Consistent with previous findings (Kelly and Conley, 1987), we found little support for the longitudinal effects of trait self-control on relationship satisfaction. However, there was one notable exception: in Study 2 we found a significant longitudinal effect, even after controlling for commitment, among pregnant women. This finding tentatively suggest that trait self-control may be particularly important during developmental transitions in a relationship, such as the transition to parenthood. During those transitions, more conscious and effortful adjustments are needed, which requires self-control (Tangney et al., 2004). Additionally, this may explain the gender differences in our findings: Women generally experience more changes (both physically and mentally) than men after the transition to parenthood (e.g., Kluwer, 2010), and they may need to exert self-control in keeping a balance between the well-being of self and the relationship. This finding may reflect, more generally, the impact of specific contexts (i.e., sample characteristics) on the role of trait self-control in romantic relationships. For example, the lack of support for the longitudinal effects of trait self-control on relationship satisfaction (and little support for partner effects, cross-sectionally) in Study 1 perhaps may be explained by the fact that this sample consisted of newlyweds. During this period, interdependence dilemmas arguably occur with lower intensity and lower frequency, and self-control therefore may be less ‘needed’ in the relationship (cf. Myrseth and Fishbach, 2009). Study 3, in which the findings more consistently provided support for the association between trait self-control and relationship satisfaction, consisted of a sample with a wider range of relationship duration, and interdependence dilemmas may have been more frequent in this sample. How contextual factors may impact the role of trait self-control in romantic relationships is an interesting topic to further explore more systematically in future studies.

As can be read in Footnote 2 (and Supplementary Material B), we also tested moderation patterns of commitment on the associations between trait self-control and relationship satisfaction. For example, one may predict that self-control is associated with relationship satisfaction only at relatively high levels of commitment (e.g., van der Wal et al., 2014). However, across the three studies, we did not find any consistent moderation between commitment and trait self-control. Interestingly, these findings may resonate with the value-based choice model of self-control (Berkman et al., 2017), which defines self-control as a process of calculating gains and costs of optional behaviors, and selecting the most highly valued behavior to enact (see also Footnote 1). People who are highly committed to the relationship may be more likely to more or less automatically select or “choose” the behavioral option that promotes the wellbeing of the partner and the relationship, resulting in higher relationship satisfaction. In terms of the value-based model of self-control, commitment provides “value” to behavioral options that promote the partner and/or the relationship, and such behaviors are thus more likely to be enacted, even without a need to exert self-control (cf., Karremans and Aarts, 2007; Righetti et al., 2013). Again, our findings seem to highlight the role of motivation (vs. ability) in relationship satisfaction.

The present research has some practical implications. Based on previous findings on the benefits of self-control in relationship outcomes, it has been suggested that promoting self-control in partners may be an effective way to increase relationship functioning and wellbeing (e.g., Finkel et al., 2009). There has been much debate about whether self-control training is feasible (Inzlicht and Berkman, 2015). Even when training programs would be effective in promoting self-control, our results raise the question of whether and how much this increased self-control would actually promote the wellbeing of a romantic relationship. The present findings suggest that the link between trait self-control and relationship satisfaction is not straightforward and robust, and self-control training as a way to improve the wellbeing of relationships therefore is not obvious (unless, perhaps, when partners suffer from clinical levels of low self-control, such as ADHD; VanderDrift et al., 2019). Instead, targeting ‘deeper’ roots of relationship distress, such as attachment- or commitment-related issues (as done in, for example, emotion-focused couple therapy; Johnson, 2012), probably is more effective in promoting relationship satisfaction.

Before closing, we should discuss several limitations. First, the samples mainly consisted of relatively happy heterosexual couples and the lack of variability in relationship satisfaction might underestimate the strength of the associations between trait self-control and relationship satisfaction (Wickham and Knee, 2012). Relatedly, the negative impact of trait self-control on relationships may only appear at ‘clinical’ levels of low self-control, probably underrepresented in our sample. Second, across the three studies, all measures were self-reported, which may inflate the correlations between variables, in particular when examining actor effects, while underestimating partner effects (Donnellan et al., 2007). More generally, the use of self-report measures limits the conclusions that can be drawn from the current research and previous studies regarding the role of self-reported trait self-control in romantic relationships. Self-reports of self-control may be biased by processes like impression management and social desirability. Moreover, among the existing approaches of self-control measurements (i.e., self-report and informant-report questionnaires, and lab tasks), self-report questionnaires tend to be moderately correlated with informant-report questionnaires, and only weakly with lab tasks (Duckworth and Kern, 2011). Thus, the current findings cannot be generalized to indicators of self-control as measured with the other two approaches. Whether and how such informant-report questionnaires and behavioral measures of self-control are associated with romantic relationship functioning remains an important issue to be further explored in future studies (see Karremans et al., 2015). Third, sample characteristics and measures were not identical between studies, which may have contributed to some inconsistent findings between studies.

In spite of these limitations, the current findings contribute to our understanding of the concurrent and longitudinal effects of trait self-control on relationship satisfaction. Is trait self-control the key to relationship success? With three independent datasets, the findings seem to provide a relatively reliable estimation of the association between trait self-control and relationship satisfaction, which was weaker and less robust than the extant literature on the role of self-control in romantic relationships would suggest.

## Data Availability Statement

The data analyzed in this study is subject to the following licenses/restrictions: The raw data supporting the conclusions of this article will be made available by the authors, if the original owner of the dataset agrees. Requests to access these datasets should be directed to P-YZ, p.zuo@psych.ru.nl.

## Ethics Statement

The studies involving human participants were conducted in compliance with the approved research and consent protocols of the Faculty of Social Sciences of the Free University of Amsterdam (Study 1), the Faculty of Social Sciences of Utrecht University (Study 2). Being more recently conducted, study 3 has received formal approval from the Ethics Committee of the Faculty of Social Sciences of Radboud University. The patients/participants provided their written informed consent to participate in this study.

## Author Contributions

P-YZ, JK, AS, and EK developed the initial research question, designed the study, revised the manuscript altogether, and approved the final version of the manuscript. HK and EK collected the data used in Study 2. GK and JK collected the data used in Study 3. P-YZ conducted the analysis and wrote the first draft of the manuscript. WB contributed to the data analysis and the improvement of the manuscript. All authors contributed to the article and approved the submitted version.

## Conflict of Interest

The authors declare that the research was conducted in the absence of any commercial or financial relationships that could be construed as a potential conflict of interest.

## Footnotes

1 It is important to mention that there is an ongoing debate about the nature of self-control and its underlying fundamental processes. For example, dual-process models of self-control regard inhibition as a fundamental aspect of self-control, allowing more controlled processes to override automatic responses (e.g., Hofmann et al., 2009). Recently, the value-based choice model of self-control (Berkman et al., 2017) provides a different view, arguing that although decisions often feel as if effortful inhibition is needed, ultimately a decision results from a dynamic integration process during which the subjective values of different behavioral options are computed. The option with the greatest value will result in action. Notably, the current research is not designed to test these specific models against each other.

2 Notably, we also explored interactions between self-control and relationship commitment on relationship satisfaction (both cross-sectionally and longitudinally). As indicated in previous literature, low levels of self-control could perhaps be compensated intra-personally by own high levels of commitment (cf. Balliet et al., 2011). Additionally, low levels of self-control might be compensated by the partner’s high levels of commitment. Finally, perhaps both high self-control and high commitment in both partners may be required to promote relationship satisfaction (i.e., a synergistic model). For the sake of brevity, however, we will not report the outcomes in the main text. In short, while we found some significant interactions in each study, we found no consistent interaction patterns across the three studies (see Supplementary Material B).

3 The original scale consists of 12 items. However, Cronbach’s alpha for the original 12-item scale was 0.67 for men and 0.47 for women, respectively. For an acceptable reliability, the final scale consists of 5 items.

4 www.flycatcher.eu
