# Supplementary material for: A Dyadic Test of the Association Between Trait Self-Control and Romantic Relationship Satisfaction
Source: Front Psychol. 2020 Dec 21;11:594476. doi: 10.3389/fpsyg.2020.594476 (PMC7779548; doi:10.3389/fpsyg.2020.594476)
Supplement: Supplementary file 1 [file Data_Sheet_1.pdf]

Supplemental Material A. APIMs Statistics in the Three Studies

Table A1

Cross-sectional APIMs on the Dyadic Associations between Trait self-control and Relationship Satisfaction at T1 (Study 1, N *= 195)*

	The basic model						The model controlling for relationship commitment at T1					
	Men			Women			Men			Women		
	b	***SE***	***p***	***b***	***SE***	***p***	***b***	***SE***	***p***	***b***	***SE***	***p***
Intercept	3.03	.20	< .001	3.17	.35	< .001	1.00	.40	.012	.57	.77	.458
**Actor effect**[a]												
T1 Trait self-control	0.30	.06	< .001	0.16	.06	.010	0.23	.04	< .001	.09	.06	.141
T1 Relationship commitment	/	/	/	/	/	/	0.46	.08	< .001	.44	.12	< .001
**Partner effect**[b]												
T1 Trait self-control	0.08	.06	.165	0.15	.08	.047	0.03	.05	.584	.10	.06	.084
T1 Relationship commitment	/	/	/	/	/	/	0.07	.05	.203	.21	.07	.002
Fit SABIC	855.44						1197.32					

Note. [a] Actor effect indicates the effect of partner A's predictor on partner A's relationship satisfaction (unstandardized regression coefficients).
[b] Partner effect indicates the effect of partner B's predictor on partner A's relationship satisfaction (unstandardized regression coefficients).

Table A2

Longitudinal APIMs on the Dyadic Associations between Trait self-control at T1 and Relationship Satisfaction at T2 (Study 1, N *= 195)*

	**The basic model**						**The model controlling for relationship commitment at T1**					
	**Men**			**Women**			**Men**			**Women**		
	b	***SE***	***p***	***b***	***SE***	***p***	***b***	***SE***	***p***	***b***	***SE***	***p***
Intercept	.99	.48	.039	1.06	.62	.085	1.05	.49	.031	1.07	.64	.093
**Actor effect**[a]												
T1 Relationship satisfaction	.73	.08	< .001	0.41	.20	.041	0.70	.10	< .001	0.41	.21	.046
T1 Trait self-control	.04	.07	.550	0.11	.07	.136	0.04	.07	.560	0.11	.08	.139
T1 Relationship commitment	/	/	/	/	/	/	0.05	.08	.563	-0.01	.09	.897
**Partner effect**[b]												
T1 Relationship satisfaction	.03	.05	.577	0.27	.10	.008	0.05	.06	.403	0.26	.15	.071
T1 Trait self-control	-.06	.07	.404	-0.05	.06	.362	-0.06	.08	.433	-0.06	.06	.357
T1 Relationship commitment	/	/	/	/	/	/	-0.05	.06	.445	0.01	.10	.887
Fit SABIC	1035.49						1385.04					

Note. As descried in the method section, all the longitudinal models in the current study controlled for both partners' relationship satisfaction at T1. The actor and partner effects refer to the effects of predictors on the *change* of relationship satisfaction between T1 and T2 (i.e., slope).

[a] Actor effect indicates the effect of partner A's predictor on partner A's relationship satisfaction (unstandardized regression coefficients).

[b] Partner effect indicates the effect of partner B's predictor on partner A's relationship satisfaction (unstandardized regression coefficients).

Table A3

Cross-sectional APIMs on the Dyadic Associations between Trait self-control and Relationship Satisfaction at T1 (Study 2, N *= 249)*

	**The basic model**						**The model controlling for relationship commitment at T1**					
	**Men**			**Women**			**Men**			**Women**		
	b	***SE***	***p***	***b***	***SE***	***p***	***b***	***SE***	***p***	***b***	***SE***	***p***
Intercept	3.15	.37	< .001	3.63	.28	< .001	-1.52	.79	.053	-3.16	1.05	.003
**Actor effect**[a]												
T1 Trait self-control	0.28	.07	< .001	0.24	.06	< .001	0.15	.05	.001	0.16	0.05	.001
T1 Relationship commitment	/	/	/	/	/	/	0.92	.12	< .001	1.16	0.18	< .001
**Partner effect**[b]												
T1 Trait self-control	0.14	.07	.038	0.04	.06	.470	0.11	.05	.044	-0.03	0.04	.432
T1 Relationship commitment	/	/	/	/	/	/	0.16	.15	.280	0.33	0.14	.021
Fit SABIC	1439.06						1342.91					

Note. [a] Actor effect indicates the effect of partner A's predictor on partner A's relationship satisfaction (unstandardized regression coefficients).
[b] Partner effect indicates the effect of partner B's predictor on partner A's relationship satisfaction (unstandardized regression coefficients).

Table A4

Longitudinal APIMs on the Dyadic Associations between Trait self-control at T1 and Relationship Satisfaction at T2 (Study 2, N *= 249)*

	The basic model						The model controlling for relationship commitment at T1					
	Men			Women			Men			Women		
	b	***SE***	***p***	***b***	***SE***	***p***	***b***	***SE***	***p***	***b***	***SE***	***p***
Intercept	-.74	.65	.259	1.07	.62	.083	-2.59	2.10	.217	-.70	1.83	.701
**Actor effect**[a]												
T1 Relationship satisfaction	.63	.12	< .001	0.54	.12	< .001	0.59	0.12	< .001	.48	0.13	< .001
T1 Trait self-control	.05	.10	.614	0.19	.08	.021	0.05	0.10	.582	.21	0.08	.010
T1 Relationship commitment	/	/	/	/	/	/	0.17	0.22	.429	.20	0.34	.548
**Partner effect**[b]												
T1 Relationship satisfaction	.44	.12	< .001	0.09	.09	.319	0.36	0.13	.005	.01	0.11	.964
T1 Trait self-control	.04	.12	.725	0.00	.09	.983	0.06	0.12	.637	.00	0.08	.966
T1 Relationship commitment	/	/	/	/	/	/	0.32	0.38	.398	.29	0.17	.093
Fit SABIC				1783.24						1691.69		

Note. As descried in the method section, all the longitudinal models in the current study controlled for both partners' relationship satisfaction at T1. The actor and partner effects refer to the effects of predictors on the *change* of relationship satisfaction between T1 and T2 (i.e., slope).

[a] Actor effect indicates the effect of partner A's predictor on partner A's relationship satisfaction (unstandardized regression coefficients).

[b] Partner effect indicates the effect of the partner B's predictor on partner A's relationship satisfaction (unstandardized regression coefficients).

Table A5

Cross-sectional APIMs on the Dyadic Associations between Trait self-control and Relationship Satisfaction (Study 3, N *= 929)*

	The basic model						The model controlling for relationship commitment					
	Men			Women			Men			Women		
	b	*SE*	*p*	*b*	*SE*	*p*	*b*	*SE*	*p*	*b*	*SE*	*p*
Intercept	4.02	.17	< .001	3.97	.18	< .001	-.03	.20	.878	-.38	.20	.056
**Actor effect**[a]												
Trait self-control	0.23	.02	< .001	0.16	.03	< .001	.08	.02	< .001	.04	.02	.028
Relationship commitment	/	/	/	/	/	/	.62	.04	< .001	.73	.03	< .001
**Partner effect**[b]												
Trait self-control	0.14	.03	< .001	0.21	.03	< .001	.05	.02	.006	.06	.02	.001
Relationship commitment	/	/	/	/	/	/	.21	.04	< .001	.16	.03	< .001
Fit SABIC	10618.74						14056.52					

Note. [a] Actor effect indicates the effect of partner A's predictor on partner A's relationship satisfaction (unstandardized regression coefficients).
[b] Partner effect indicates the effect of the partner B's predictor on partner A's relationship satisfaction (unstandardized regression coefficients).

Supplemental Material B. APIMoMs and Simple Slope Test Statistics with Relationship Commitment as the Moderator

In addition to investigate the dyadic relationship between trait self-control and relationship satisfaction, we explored interactions between trait self-control and relationship commitment on relationship satisfaction (both cross-sectionally and longitudinally).

Method

Actor–partner interdependence moderation models (APIMoMs) with structural equation modeling using the Lavaan package (Rosseel, 2012) in R was applied. Since we have a mixed moderator (i.e., relationship commitment) among distinguishable dyads (i.e., heterosexual couples), we explored the potential moderating effects with the corresponding strategy as proposed by Garcia, Kenny, and Ledermann (2015). Separate APIMoMs were estimated for the moderator (i.e., relationship commitment). Both partners' trait self-control, and relationship commitment at T1 were predictive variables, that were grand-mean-centered (i.e., centered at the couple's mean value of the corresponding variable). Both partners' relationship satisfaction at T1 were outcome variables in all the cross-sectional APIMoMs, and were controlling variables for longitudinal APIMoMs. Both partners' relationship satisfaction at T2 were outcome variables in all the longitudinal APIMoMs. For saturated models, the sampling-error-adjusted Bayesian information criterion (SABIC) was the model fit index (Garcia et al., 2015). For unsaturated models, we applied the following fit indices to evaluate the models (Hooper et., 2008): The Absolute Fit Indices Chi-Square (χ^2) test should yield low χ^2 relative to degrees of freedom with an insignificant *p* value ($p > .05$), the comparative fit index (CFI) should be larger than .95, and the root mean square error of approximation (RMSEA) should be less than .05. For all the significant interactions, simple slopes were calculated and plotted at ±1 *SD* from the mean and at the mean.

For all three studies, the same data analysis strategy was used for the moderator (i.e., relationship commitment).

Study 1

Results and Discussion

Detailed APIMoMs statistics on the potential moderating role of relationship commitment are presented in Table B1, B2, B3 and B9. A number of significant interactions were found (see Table B3 and Figure 1).

First, own commitment interacted with own self-control in predicting own concurrent relationship satisfaction for both men ($b = -.21$, $SE = .10$, $p = .041$) and women ($b = -.49$, $SE = .20$, $p = .015$). As can be seen in Figure 1A (men) and 1B (women), the positive association between own self-control and own concurrent satisfaction was strongest at low levels of commitment for both men ($b = .31$, $t = 4.79$, $p = .008$) and women ($b = .32$, $t = 3.21$, $p = .002$). Among highly committed partners, the association was weaker for men ($b = .13$, $t = 2.53$, $p = .012$), and non-significant for women ($b = -.09$, $t = -.88$, $p = .379$). The results indicated that high level of relationship commitment could moderate the negative effects of one's own low self-control on own relationship satisfaction for both genders (esp., for women).

Second, men's commitment ($b = .21$, $SE = .10$, $p = .041$) interacted with women's self-control in predicting men's concurrent satisfaction. Among men high in commitment ($b = .13$,

$t = 2.30$, $p = .022$; but not medium and low; medium, $b = .04$, $t = .87$; low, $b = -.05$, $t = -.75$, $ps > .383$), there was a significant partner effect such that the women's self-control was positively associated with the men's concurrent satisfaction (see Figure 1C).

Third, women's commitment (Figure 1, D; $b = -.39$, $SE = .19$, $p = .038$) interacted with women's self-control in predicting the male partner's satisfaction 3 years' later. Among women high in commitment ($b = -.19$, $t = -2.26$, $p = .025$; but not medium and low; medium, $b = -.03$, $t = -.35$; low, $b = .13$, $t = 1.05$, $ps > .294$), there was, somewhat surprisingly, a significant *negative* partner effect such that the women's self-control was negatively associated with the male partner's relationship satisfaction 3 years' later.

In sum, we found some support for the moderation role of relationship commitment on the associations between trait self-control and relationships satisfaction, but without any consistent moderating pattern between genders, nor between cross-sectional and longitudinal models.

Study 2

Results and Discussion

In Study 2, we again examined the potential moderating effects of relationship commitment with APIMoMs. Detailed statistics are presented in Table B4, B5, B6 and B9. A number of significant interactions were found, but only on men's relationship satisfaction (see Table B6, & Figure 2).

First, men's commitment ($b = -.29$, $SE = .08$, $p < .001$) interacted with men's own self-control in predicting own concurrent relationship satisfaction, which is consistent with the findings in Study 1. As can be seen in Figures 2A, the positive association between men's own self-control and own concurrent satisfaction was strongest at low levels of men's commitment ($b = .26$, $t = 4.61$, $p = .016$). The association was weaker among men with medium level of commitment ($b = .16$, $t = 3.63$, $p < .001$), but was not significant among highly committed men ($b = .07$, $t = 1.39$, $p = .165$).

Second, women's commitment interacted with women's self-control in predicting the male partner's (but not women's own) concurrent satisfaction (Figure 2, B; $b = .29$, $SE = .08$, $p < .001$), which is different from Study 1. The positive association between women's self-control and the male partner's concurrent satisfaction was strongest when women was highly committed ($b = .16$, $t = 2.97$, $p = .003$). This association was marginally significant at the women medium level of commitment ($b = .10$, $t = 1.92$, $p = .056$), and not significant when the women was low in commitment ($b = .04$, $t = .67$, $p = .505$).

Third, men's commitment interacted with the female partner's self-control ($b = -.29$, $SE = .08$, $p < .001$) in predicting men's concurrent satisfaction, but the pattern is different from the findings in Study 1. Specifically, as can be seen in the Figure 2C, the positive association between women's self-control and men's concurrent satisfaction was strongest at low (but not high) levels of women's relationship commitment ($b = .19$, $t = 3.34$, $p < .001$). The association was marginally significant among medium committed women partners ($b = .10$, $t = 1.92$, $p = .056$), but not significant among women high in commitment ($b = .00$, $t = .08$, $p = .938$). Thus, the results Study 2 also indicated an interaction between women's self-control and men's relationship commitment on men's concurrent satisfaction as Study 1, but with a different pattern (a negative but not a positive interaction).

Four, women's commitment interacted with men's self-control ($b = .29$, $SE = .08$, $p < .001$) in predicting men's concurrent relationship satisfaction. As can be seen in the Figure 2D, the positive association between men's own self-control and own concurrent satisfaction was strongest at high level of female partner's relationship commitment ($b = .22$, $t = 4.44$, $p = .009$), weaker when with a medium committed female partner ($b = .16$, $t = 3.63$, $p < .001$), and was weakest when with a low committed female partner ($b = .10$, $t = 2.19$, $p = .029$).

Five, women's commitment also interacted with women's self-control in predicting men's satisfaction 1.5 years' later (Figure 2, E; $b = -1.34$, $SE = .60$, $p = .026$), which is similar to the findings in Study 1. However, the pattern was different, as the simple slope test suggested no significant association between women's self-control and their male partner's satisfaction 1.5 years' later was found on different levels of women's commitment (ps > .077).

In sum, the results suggested a number of significant interactions between relationship commitment and trait self-control, but only on men's relationship satisfaction. When comparing with the results of Study 1, even though several similar interactions emerged, there was no consistent moderating pattern neither cross-sectionally nor longitudinally.

Study 3

Results and Discussion

In Study 3, we again examined the potential moderating effects of relationship commitment with APIMoMs. Detailed statistics are presented in Table B7, B8 and B9. As can be seen in Figure 2F, only one significant interaction emerged (see Table B8). That is, men's own commitment interacted with own self-control in predicting own concurrent satisfaction ($b = .06$, $SE = .03$, $p = .024$), which is similar to the findings in Study 1 and 2. However, the pattern was different: the positive association between men's own self-control and own concurrent satisfaction was strongest at high levels of men's own commitment ($b = .13$, $t = 5.28$, $p = .001$). The association was weaker among men medium in commitment ($b = .08$, $t = 4.46$, $p = .023$), but was non-significant among men low in commitment ($b = .03$, $t = .96$, $p = .337$). These findings are in contrast to some of the findings in Studies 1 and 2, where we found that the association was in fact *weaker* at high levels of men's own commitment.

In sum, across the three studies, the explorative analysis suggested that relationship commitment moderated the effects of trait self-control on relationship satisfaction (particularly on men's relationship satisfaction, but not women's). However, no consistent moderating pattern of commitment were identified across studies. Given that the explorative nature of the analysis, as well as the limited power for the analysis in Studies 1 and 2, we consider it as premature to speculate on those findings.

Table B1

Estimates and Fit Statistics of APIMoMs with Trait Self-control and Relationship Commitment Predicting Relationship Satisfaction at T1 (Study 1, N *= 195, Cross-sectional)*

		Effects								Fit					
**APIMoMs**		**Actor X by actor M (Me)**	**Actor X by actor M (Wo)**	**Actor X by partner M (Me)**	**Actor X by partner M (Wo)**	**Partner X by actor M (Me)**	**Partner X by actor M (Wo)**	**Partner X by partner M (Me)**	**Partner X by partner M (Wo)**	χ^2	***df***	***p***	**SABIC**	**Robust CFI**	**Robust RMSEA**
Model 1: Unrestricted		-.16	-.54*	.13	.17	.27*	-.12	.14	.00	/	0	/	826.43	1.000	.000
Model 2: Interaction effects zero		.00	.00	.00	.00	.00	.00	.00	.00	21.99	8	.005	833.80	0.913	.099
Model 3: Indistinguishable interaction effects		-.25*	-.25*	.13	.13	.15	.15	.06	.06	10.50	4	.033	826.57	0.970	.082
**Combined model 1**[a] (**The final model**: me, contrast X, actor M; wo, actor X actor M)		-.21*	-.49*	.00	.00	.21*	.00	.00	.00	6.02	6	.421	819.37	1.000	.004
**Combined model 2** (Me, partner X, actor M; wo, actor X, actor M)		.00	-.48*	.00	.00	.32*	.00	.00	.00	6.37	6	.383	820.60	0.998	.018
**M models**															
Me	Actor M only	-.14	-.56**	.00	.17	.30*	-.13	.00	.00	3.02	2	.221	824.62	0.995	.046
	Partner M only	.00	-.53*	.11	.10	.00	-.12	.22	.04	9.90	2	.007	834.20	0.946	.157
	Couple M	-.03	-.53*	-.03	.16	.23	-.14	.23	.03	6.30	2	.043	826.76	0.983	.089
	Contrast M	-.17	-.57**	.17	.13	.13	-.12	-.13	-.00	9.57	2	.008	829.91	0.966	.125
Wo	Actor M only	-.16	-.48*	.13	.00	.25	-.12	.14	.00	1.48	2	.477	823.84	1.000	.000
	Partner M only	-.16	.00	.14	.06	.27*	.00	.16	-.03	11.77	2	.003	830.45	0.962	.132
	Couple M	-.17	-.10	.13	-.10	.24	-.08	.15	-.08	7.41	2	.025	829.31	0.971	.115
	Contrast M	-.15	-.28*	.13	.28*	.30*	-.07	.15	.07	4.97	2	.083	825.83	0.988	.074
**X models**															
Me	Actor X only	-.23	-.56**	.13	.10	.00	-.12	.00	-.02	14.97	2	.001	831.33	0.956	.142
	Partner X only	.00	-.54*	.00	.18	.32*	-.13	.12	.04	4.56	2	.102	825.80	0.989	.072
	Couple X	.04	-.54*	.15*	.11	.04	-.11	.15*	.05	9.41	2	.009	834.45	0.946	.157
	Contrast X	-.22*	-.56**	.02	.15	.22*	-.13	-.02	-.02	4.28	2	.118	825.54	0.990	.067
Wo	Actor X only	-.16	-.54*	.14	.17	.27*	.00	.14	.00	0.53	2	.769	822.83	1.000	.000
	Partner X only	-.16	.00	.13	.00	.26*	-.10	.16	-.04	10.40	2	.006	830.24	0.964	.129
	Couple X	-.15	-.28	.12	.06	.26*	-.28	.15	.06	4.48	2	.107	825.77	0.989	.071
	Contrast X	-.16	-.14	.14	.06	.27*	.14	.15	-.06	8.16	2	.017	829.06	0.971	.115

Note. X = Trait self-control at T1. M = Relationship commitment at T1 (Moderator). Men = Me. Wo = Women.
[a] The combined models in the moderation analysis were based on all the X models and M models (i.e., the 16 models below). The final model was the one with a better model fit (i.e., with a smaller SABIC).
$^{*}p$ < .05. $^{**}p$ < .01.

Table B2

Estimates and Fit Statistics of APIMoMs with Trait Self-control and Relationship Commitment Predicting Relationship Satisfaction at T2 (Study 1, N *= 195, Longitudinal)*

**APIMoMs**		**Effects**								**Fit**					
		**Actor X by actor M (Me)**	**Actor X by actor M (Wo)**	**Actor X by partner M (Me)**	**Actor X by partner M (Wo)**	**Partner X by actor M (Me)**	**Partner X by actor M (Wo)**	**Partner X by partner M (Me)**	**Partner X by partner M (Wo)**	χ^2	***df***	***p***	**SABIC**	**Robust CFI**	**Robust RMSEA**
Model 1: Unrestricted		.09	.02	-.20	-.25	.09	-.2	-.45*	.00	/	0	/	1020.18	1.000	.000
Model 2: Interaction effects zero		.00	.00	.00	.00	.00	.00	.00	.00	11.09	8	.197	1014.14	0.978	.044
Model 3: Indistinguishable interaction effects		.04	.04	-.23	-.23	-.05	-.05	-.16	-.16	7.14	4	.129	1016.70	0.984	.053
**Combined model 1**[a] (Me, couple X, partner M; wo, actor X, partner M)		.00	.00	-.25	-.25	.00	.00	-.25	.00	3.42	6	.754	1011.14	1.000	.000
**Combined model 2** (**The final model**): me, partner X, partner M; wo, actor X, partner M)		.00	.00	.00	-.26	.00	.00	-.39*	.00	2.79	6	.835	1010.66	1.000	.000
**M models**															
Me	Actor M only	.04	.16	.00	-.29	-.03	-.14	.00	-.02	10.65	2	.005	1022.78	0.959	.119
	Partner M only	.00	.03	-.18	-.27	.00	-.20	-.40*	-.03	0.64	2	.725	1016.71	1.000	.000
	Couple M	-.05	.11	-.05	-.31	-.13	-.15	-.13	-.05	5.84	2	.054	1020.83	0.976	.091
	Contrast M	.14	.09	-.14	-.21	.20	-.18	-.20	.01	3.15	2	.207	1019.59	0.990	.058
Wo	Actor M only	.09	-.12	-.20	.00	.14	-.22	-.47	.00	3.22	2	.200	1018.37	0.993	.048
	Partner M only	.09	.00	-.16	-.25	.09	.00	-.45*	-.02	1.50	2	.472	1017.17	1.000	.000
	Couple M	.08	-.16	-.18	-.16	.11	-.07	-.48*	-.07	2.38	2	.304	1017.55	0.998	.025
	Contrast M	.11	.18	-.18	-.18	.10	-.07	-.41*	.07	1.58	2	.455	1017.70	1.000	.000
**X models**															
Me	Actor X only	.06	.16	-.16	-.28	.00	-.19	.00	-.01	7.16	2	.028	1021.66	0.970	.103
	Partner X only	.00	.03	.00	-.26	.05	-.14	-.41*	-.03	1.98	2	.372	1018.20	1.000	.000
	Couple X	.07	.07	-.29*	-.25	.07	-.23	-.29*	-.01	1.36	2	.506	1017.27	1.000	.000
	Contrast X	.03	.14	.05	-.28	-.03	-.12	-.05	-.02	6.69	2	.035	1022.58	0.966	.109
Wo	Actor X only	.10	.04	-.16	-.25	.09	.00	-.44*	.00	1.21	2	.546	1017.17	1.000	.000
	Partner X only	.10	.00	-.20	.00	.15	-.21	-.45*	.05	3.08	2	.215	1018.51	0.993	.048
	Couple X	.07	-.14	-.19	-.09	.12	-.14	-.48*	-.09	3.70	2	.157	1017.91	0.993	.048
	Contrast X	.12	.11	-.18	-.12	.12	-.11	-.43*	.12	1.79	2	.409	1017.97	1.000	.000

Note. X = Self-control at T1. M = Relationship commitment at T1 (Moderator). Men = Me. Wo = Women.

[a] The combined models in the moderation analysis were based on all the X models and M models (i.e., the 16 models below). The final model was the one with a better model fit (i.e., with a smaller SABIC).

$^{*}p < .05$.

Table B3

The Best Fitting APIMoMs with Trait Self-control and Relationship Commitment Predicting Relationship Satisfaction (Study 1, N *= 195, Cross-sectional & Longitudinal)*

		**Cross-sectional (Men, contrast X, actor M; Women, actor X, actor M)**			**Longitudinal (Men, partner X, partner M; Women, actor X, partner M)**		
		b	***SE***	***p***	***b***	***SE***	***p***
**Intercept**							
Men		4.28	.02	<.001	1.03	.47	.028
Women		4.17	.03	<.001	1.05	.62	.088
**Actor effect**[a]							
T1 relationship satisfaction	Men	/	/	/	0.74	.10	<.001
	Women	/	/	/	0.41	.20	.043
T1 self-control (X)	Men	0.22	.04	<.001	0.04	.07	.584
	Women	0.13	.05	.014	0.09	.07	.188
T1 relationship commitment (M)	Men	0.45	.06	<.001	0.03	.08	.667
	Women	0.39	.11	<.001	-0.02	.09	.854
**Partner effect**[b]							
T1 relationship satisfaction	Men	/	/	/	0.00	.06	.997
	Women	/	/	/	0.32	.16	.038
T1 self-control (X)	Men	0.05	.05	.304	-0.01	.08	.853
	Women	0.09	.05	.103	-0.06	.06	.299
T1 relationship commitment (M)	Men	0.07	.06	.223	-0.06	.06	.305
	Women	0.20	.07	.002	0.00	.10	.971
**Interaction effect: Actor X by actor M**							
Men		-0.21	.10	.041	/	/	/
Women		-0.49	.20	.015	/	/	/
**Interaction effect: Actor X by partner M**							
Men		/	/	/	/	/	/
Women		/	/	/	-0.26	.15	.093
**Interaction effect: Partner X by actor M**							
Men		0.21	.10	.041	/	/	/
Women		/	/	/	/	/	/
**Interaction effect: Partner X by partner M**							
Men		/	/	/	-0.39	.19	.038
Women		/	/	/	/	/	/

Note. M = Moderator. Y = Relationship satisfaction at T1 (cross-sectional model) or T2 (longitudinal model).

[a] Actor effect indicates the effect of partner A's predictor on partner A's relationship satisfaction (unstandardized regression coefficients).

[b] Partner effect indicates the effect of partner B's predictor on partner A's relationship satisfaction (unstandardized regression coefficients).

Table B4

Estimates and Fit statistics of APIMoMs with Trait Self-control and Relationship Commitment Predicting Relationship Satisfaction at T1 (Study 2, N *= 249, Cross-sectional)*

	Effects								Fit					
**APIMoMs**	**Actor X by actor M (Me)**	**Actor X by actor M (Wo)**	**Actor X by partner M (Me)**	**Actor X by partner M (Wo)**	**Partner X by actor M (Me)**	**Partner X by actor M (Wo)**	**Partner X by partner M (Me)**	**Partner X by partner M (Wo)**	χ^2	***df***	***p***	**SABIC**	**Robust CFI**	**Robust RMSEA**
Model 1: Unrestricted	-.30	-.17	.28	.18	-.31[a]	.01	.17	.02	/	0	/	299.64	1.000	.000
Model 2: Interaction effects zero	.00	.00	.00	.00	.00	.00	.00	.00	14.21	8	.076	297.43	0.971	.060
Model 3: Indistinguishable interaction effects	-.29	-.29	.21	.21	-.20	-.20	.09	.09	1.85	4	.763	292.14	1.000	.000
**The final model**[a]:														
Indistinguishable couple X contrast M	$-.29^{***}$	-.10	$.29^{***}$	.10	$-.29^{***}$	-.10	$.29^{***}$	.10	0.79	6	.992	286.46	1.000	.000
**M models**														
Actor M only	-.24	-.18	.00	.00	$-.36^{*}$	-.04	.00	.00	2.49	4	.646	294.46	1.000	.000
Partner M only	.00	.00	.15	.26	.00	.00	.12	.10	16.09	4	.003	301.72	0.966	.093
Couple M	-.06	.09	-.06	.09	-.24[b]	.03	-.24[b]	.03	8.21	4	.084	300.02	0.980	.071
Contrast M	$-.30^{*}$	-.18	$.30^{*}$	.18	-.29	-.01	.29	.01	0.29	4	.990	290.60	1.000	.000
**X models**														
Actor X only	$-.42^{**}$	-.22	.29	.27	.00	.00	.00	.00	3.07	4	.546	294.37	1.000	.000
Partner X only	.00	.00	.00	.00	$-.44^{**}$	-.08	.23	.17	4.52	4	.341	295.21	0.998	.024
Couple X	$-.31^{***}$	-.06	.24	.11	$-.31^{***}$	-.06	.24	.11	0.53	4	.970	290.83	1.000	.000
Contrast X	.06	-.09	.06	.10	-.06	.09	-.06	-.10	13.66	4	.008	305.62	0.957	.104

Note. X = Self-control at T1. M = Relationship commitment at T1 (Moderator). Men = Me. Wo = Women.

[a] The final model was a combined model that was based on all the X models and M models (i.e., the 8 models below).

$^{*}p < .05$. $^{**}p < .01$. $^{***}p < .001$. $^{a}p = .056$. $^{b}p = .052$.

Table B5

Estimates and Fit statistics of APIMoMs with Trait Self-control and Relationship Commitment Predicting Relationship Satisfaction at T2 (Study 2, N *= 249, Longitudinal)*

		Effects								Fit					
**APIMoMs**		**Actor X by actor M (Me)**	**Actor X by actor M (Wo)**	**Actor X by partner M (Me)**	**Actor X by partner M (Wo)**	**Partner X by actor M (Me)**	**Partner X by actor M (Wo)**	**Partner X by partner M (Me)**	**Partner X by partner M (Wo)**	χ^2	***df***	***p***	**SABIC**	**Robust CFI**	**Robust RMSEA**
Model 1-Unrestricted		.37	-.34	-.35	-.51	-.04	-.79	-1.75*	.46	/	0	/	647.90	1.000	.000
Model 2-Interaction effects zero		.00	.00	.00	.00	.00	.00	0.00	.00	22.23	8	.005	648.29	0.916	.078
Model 3-Indistinguishable interaction effects		.39	.39	-.32	-.32	-.40	-.40	0.04	.04	20.12	4	.000	651.95	0.926	.104
**The final model**[a]: men, partner X, partner M; women, contrast X, contrast M		.00	.43	.00	-.43	.00	-.43	-1.34*	.43	5.46	6	.486	639.94	1.000	.000
**M models**															
Me	Actor M only	.34	.33	.00	-.58	-.24	-.66	0.00	.44	7.85	2	.020	652.00	0.955	.115
	Partner M only	.00	-.31	-.16	-.50	.00	-.72	-1.70*	.32	1.88	2	.391	645.19	1.000	.000
	Couple M	.20	.18	.20	-.65	-.39	-.58	-0.39	.39	8.58	2	.014	650.79	0.960	.108
	Contrast M	.19	.23	-.19	-.40	.27	-.74[a]	-0.27	.39	8.71	2	.013	651.36	0.957	.112
Wo	Actor M only	.16	-.50	-.29	.00	.20	-.67	-1.82*	.00	3.72	2	.156	647.15	0.987	.061
	Partner M only	.28	.00	.03	-.54	-.05	.00	-1.58**	.25	4.97	2	.083	649.33	0.975	.086
	Couple M	.12	-.31	-.01	-.31	.06	-.07	-1.72**	-.07	8.39	2	.015	649.87	0.965	.101
	Contrast M	.39	.49	-.21	-.49	-.03	-.50	-1.38*	.50	2.29	2	.318	646.76	0.997	.030
**X models**															
Me	Actor X only	.29	.33	-.22	-.50	.00	-.75	0.00	.43	19.12	2	.000	652.13	0.945	.127
	Partner X only	.00	-.33	.00	-.45	.14	-.65	-1.73*	.32	1.61	2	.446	645.17	1.000	.000
	Couple X	.20	.10	-.64	-.43	.20	-.91[b]	-0.64	.40	13.12	2	.001	649.30	0.965	.102
	Contrast X	.24	.23	.26	-.59	-.24	-.56	-0.26	.40	6.48	2	.039	651.35	0.961	.106
Wo	Actor X only	.16	-.29	.03	-.36[a]	.03	.00	-1.71*	.00	7.05	2	.029	650.05	0.966	.099
	Partner X only	.27	.00	-.34	.00	.20	-.78	-1.60**	.22	2.61	2	.271	647.08	0.994	.043
	Couple X	.17	-.64	-.28	.01	.20	-.64	-1.89*	.01	3.31	2	.191	647.22	0.989	.056
	Contrast X	.38	.52	-.22	-.48	-.02	-.52	-1.36*	.48	2.75	2	.253	646.74	0.993	.044

Note. X = Trait self-control at T1. M = Relationship commitment at T1 (Moderator). Men = Me. Wo = Women.

[a] The final model was a combined model that was based on all the X models and M models (i.e., the 16 models below).

*$p < .05$. **$p < .01$. [a]$p = .050$. [b]$p = .056$.

Table B6

The Best Fitting APIMoMs with Trait Self-control and Relationship Commitment Predicting Relationship Satisfaction at T1 and T2 (Study 2, N *= 249)*

		**Relationship satisfaction at T1 (Indistinguishable couple X contrast M)**			**Relationship satisfaction at T2 (Men: partner X, partner M; women: contrast X, contrast M)**		
		b	***SE***	***p***	***b***	***SE***	***p***
**Intercept**							
Men		4.51	.03	< .001	0.24	.71	.742
Women		4.50	.03	< .001	2.21	.54	< .001
**Actor effect**[a]							
T1 Relationship satisfaction	Men	/	/	/	0.62	.12	< .001
	Women	/	/	/	0.49	.13	< .001
T1 Trait self-control	Men	0.15	.04	.001	0.02	.09	.795
	Women	0.17	.05	.001	0.17	.08	.039
T1 Relationship commitment (M)	Men	0.77	.11	< .001	0.09	.22	.673
	Women	1.15	.18	< .001	0.31	.28	.273
**Partner effect**[b]							
T1 Relationship satisfaction	Men	/	/	/	0.31	.14	.022
	Women	/	/	/	0.01	.10	.935
T1 Trait self-control	Men	0.09	.05	.084	0.14	.13	.306
	Women	-0.03	.04	.430	0.02	.08	.796
T1 Relationship commitment (M)	Men	0.22	.14	.13	0.02	.33	.961
	Women	0.38	.14	.005	0.45	.23	.051
**Interaction effect: Actor X by actor M**							
Men		-0.29	.08	< .001	/	/	/
Women		-0.10	.15	.498	0.43	.24	.076
**Interaction effect: Actor X by partner M**							
Men		0.29	.08	< .001	/	/	/
Women		0.10	.15	.498	-0.43	.24	.076
**Interaction effect: Partner X by actor M**							
Men		-0.29	.08	< .001	/	/	/
Women		-0.10	.15	.498	-0.43	.24	.076
**Interaction effect: Partner X by partner M**							
Men		0.29	.08	< .001	-1.34	.60	.026
Women		0.10	.15	.498	0.43	.24	.076

Note. M = Moderator.

[a] Actor effect indicates the effect of partner A's predictor on partner A's relationship satisfaction (unstandardized regression coefficients).

[b] Partner effect indicates the effect of partner B's predictor on partner A's relationship satisfaction (unstandardized regression coefficients).

Table B7

Estimates and Fit Statistics of APIMoMs with Trait Self-control and Relationship Commitment Predicting Relationship Satisfaction at T1 (Study 3, N *= 929, Cross-sectional)*

	Effects								Fit					
**APIMoMs**	**Actor X by actor M (Me)**	**Actor X by actor M (Wo)**	**Actor X by partner M (Me)**	**Actor X by partner M (Wo)**	**Partner X by actor M (Me)**	**Partner X by actor M (Wo)**	**Partner X by partner M (Me)**	**Partner X by partner M (Wo)**	χ^2	***df***	***p***	**SABIC**	**Robust CFI**	**Robust RMSEA**
Model 1: Unrestricted	.09**	.05	-.03	-.02	-.03	.02	.04	.04	/	0	/	25020.94	1.000	.000
Model 2: Interaction effects zero	.00	.00	.00	.00	.00	.00	.00	.00	18.60	8	.017	25018.08	0.992	.045
Model 3: Indistinguishable interaction effects	.07**	.07**	-.03	-.03	-.01	-.01	.04*	.04*	3.13	4	.536	25010.62	1.000	.000
**The final model**[a]: Indistinguishable actor X actor M	.06*	.04	.00	.00	.00	.00	.00	.00	9.29	6	.158	25011.98	0.998	.029
**M models**														
Actor M only	.06*	.03	.00	.00	.00	.04[a]	.00	.00	5.65	4	.227	25014.13	0.999	.025
Partner M only	.00	.00	.00	.02	.00	.00	.02	.02	14.09	4	.007	25029.55	0.991	.067
Couple M	.03*	.01	.03*	.01	.01	.03*	.01	.03*	8.29	4	.082	25021.27	0.996	.046
Contrast M	.05*	.03	-.05*	-.03	-.03	-.01	.03	.01	14.19	4	.007	25019.60	0.995	.051
**X models**														
Actor X only	.07*	.04	-.03	.00	.00	.00	.00	.00	7.46	4	.113	25016.18	0.998	.035
Partner X only	.00	.00	.00	.00	-.01	.03	.02	.01	18.04	4	.001	25027.62	0.991	.067
Couple X	.03	.03	.00	.01	.03	.03	.00	.01	11.73	4	.019	25019.47	0.995	.048
Contrast X	.05*	.02	-.03*	-.03	-.05*	-.02	.03*	.03	8.78	4	.067	25020.92	0.996	.046

Note. X = Trait self-control. M = Relationship commitment (Moderator). Men = Me. Wo = Women.
[a] The final model was a combined model that was based on all the X models and M models (i.e., the 8 models below).
$^{*}p < .05$. $^{**}p < .01$. $^{a}p = .050$.

Table B8

The Best Fitting APIMoM with Trait Self-control and Relationship Commitment Predicting Relationship Satisfaction at T1 (Study 3, N *= 929)*

		**Relationship satisfaction at T1 (Indistinguishable actor X actor M)**		
		b	***SE***	***p***
**Intercept**				
Men		5.82	.02	< .001
Women		5.73	.02	< .001
**Actor effect**[a]				
T1 Trait self-control	Men	0.08	.02	< .001
	Women	0.04	.02	.043
T1 Relationship commitment (M)	Men	0.65	.04	< .001
	Women	0.75	.03	< .001
**Partner effect**[b]				
T1 Trait self-control	Men	0.05	.02	.004
	Women	0.06	.02	.001
T1 Relationship commitment (M)	Men	0.20	.03	< .001
	Women	0.16	.03	< .001
**Interaction effect: Actor X by actor M**				
Men		0.06	.03	.024
Women		0.04	.02	.083
**Interaction effect: Actor X by partner M**				
Men		/	/	/
Women		/	/	/
**Interaction effect: Partner X by actor M**				
Men		/	/	/
Women		/	/	/
**Interaction effect: Partner X by partner M**				
Men		/	/	/
Women		/	/	/

Note. M = Moderator.

[a] Actor effect indicates the effect of partner A's predictor on partner A's relationship satisfaction (unstandardized regression coefficients).

[b] Partner effect indicates the effect of partner B's predictor on partner A's relationship satisfaction (unstandardized regression coefficients).

Table B9

The Moderation Effects of Relationship Commitment on the Dyadic Associations between Trait Self-control and Relationship Satisfaction

Studies	Gender	The pattern of the moderation	Significant interactions	The results of simple slopes test on each level of relationship commitment	
				Significant simple slopes	Non-significant simple slopes
**Cross-sectional models**					
Study 1	Men	Contrast X, actor M	Actor X by actor M (m*m)	m*m: High ($b = .13$, $t = 2.53$, $p = .012$)<br>Medium ($b = .22$, $t = 5.73$, $p = .001$)<br>Low ($b = .31$, $t = 4.79$, $p = .008$)	/
			Partner X by actor M (w*m)	w*m: High ($b = .13$, $t = 2.30$, $p = .022$)	w*m: Medium ($b = .04$, $t = .87$, $p = .384$)<br>Low ($b = -.05$, $t = -.75$, $p = .457$)
	Women	Actor X, actor M	Actor X by actor M (w*w)	w*w: Medium ($b = .12$, $t = 2.19$, $p = .030$)<br>Low ($b = .32$, $t = 3.21$, $p = .002$)	w*w: High ($b = -.09$, $t = -.88$, $p = .379$)
Study 2	Men	Indistinguishable couple X contrast M	Actor X by actor M (m*m)	m*m: Medium ($b = .16$, $t = 3.63$, $p < .001$)<br>Low ($b = .26$, $t = 4.61$, $p = .016$)	m*m: High ($b = .07$, $t = 1.39$, $p = .165$)
			Actor X by partner M (m*w)	m*w: High ($b = .22$, $t = 4.44$, $p = .009$)<br>Medium ($b = .16$, $t = 3.63$, $p < .001$)<br>Low ($b = .10$, $t = 2.19$, $p = .029$)	/
			Partner X by partner M (w*w)	w*w: High ($b = .16$, $t = 2.97$, $p = .003$)<br>Medium ($b = .10$, $t = 1.92$, $p = .056$)	w*w: Low ($b = .04$, $t = .67$, $p = .505$)
			Partner X by actor M (w*m)	w*m: Medium ($b = .10$, $t = 1.92$, $p = .056$)<br>Low ($b = .19$, $t = 3.34$, $p < .001$)	w*m: High ($b = .00$, $t = .08$, $p = .938$)
	Women		/	/	/
Study 3	Men	Indistinguishable actor X actor M	Actor X, Actor M (m*m)	m*m: High ($b = .13$, $t = 5.28$, $p = .001$)<br>Medium ($b = .08$, $t = 4.46$, $p = .023$)	m*m: Low ($b = .03$, $t = .96$, $p = .337$)
	Women		/	/	/
**Longitudinal models**					
Study 1	Men	Partner X, partner M	Partner X, Partner M (w*w)	w*w: High ($b = -.19$, $t = -2.26$, $p = .025$)	w*w: Medium ($b = -.03$, $t = -.35$, $p = .725$)<br>Low ($b = .13$, $t = 1.05$, $p = .295$)
	Women	Actor X, partner M	/	/	/
Study 2	Men	Partner X, partner M	Partner X, Partner M (w*w)	/	/
	Women	Contrast X, contrast M	/	/	/

Note. w = women. m = men.

Figure 1

The Moderating Effects of Relationship Commitment on the Association between Trait Self-control and Relationship Satisfaction (Study 1)

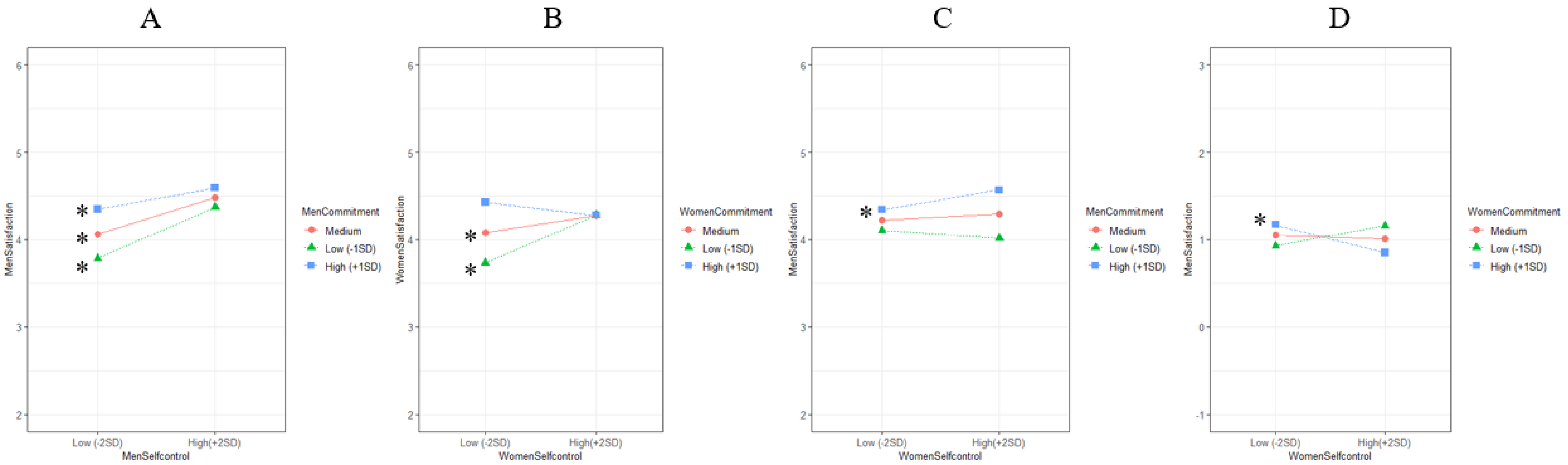

Note. A-C: Cross-sectional effects. D: Longitudinal effects. "*" indicates the significance level of the simple slope test (i.e., whether the simple slope is significantly different from zero). A: $^{*}ps < .012$. B: $^{*}p < .030$. C: $^{*}p = .022$. D: $^{*}p = .025$.

Figure 2

The Moderating Effects of Relationship Commitment on the Association between Trait Self-control and Relationship Satisfaction (Studies 2 & 3)

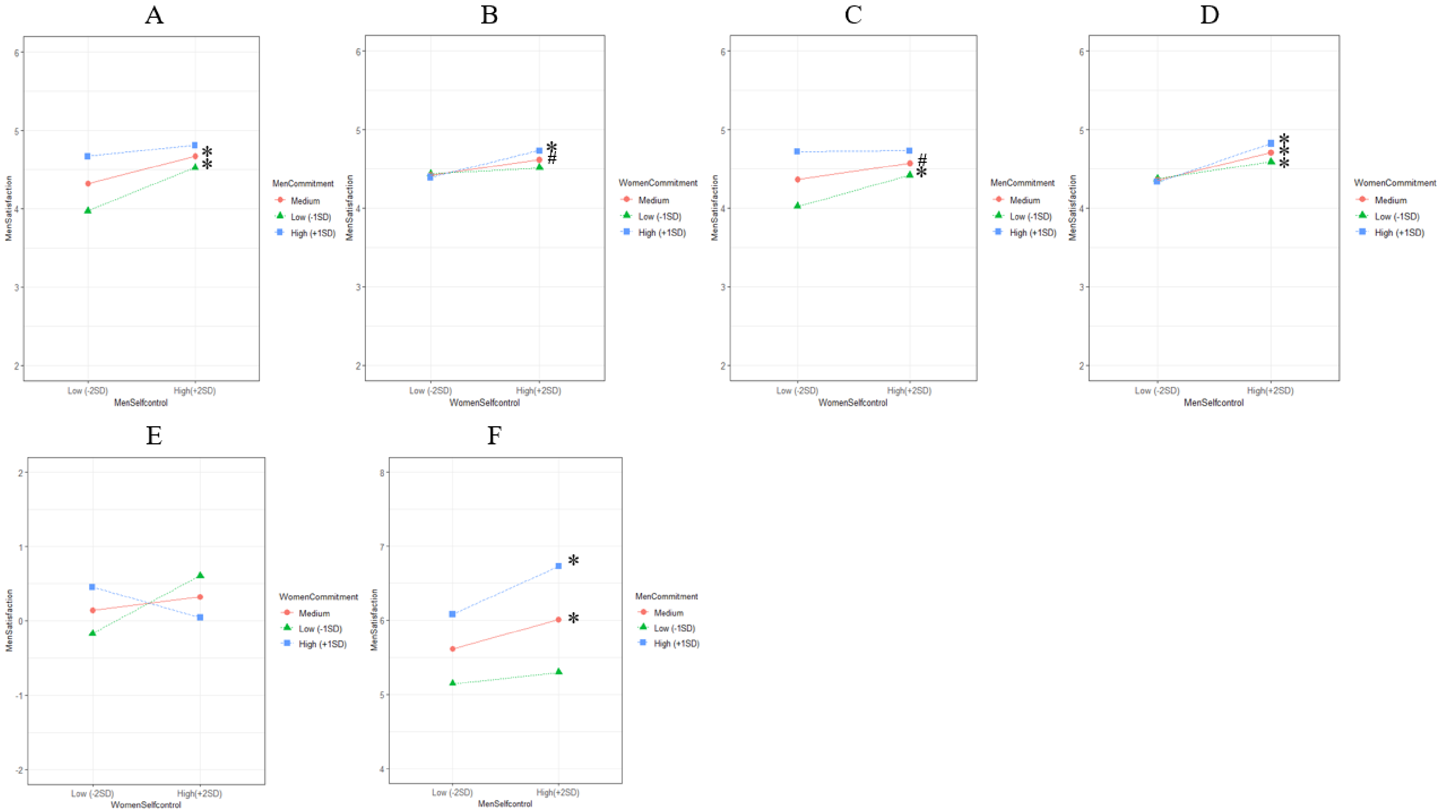

Note. A-E: Study 2 (A-D: cross-sectional effects; E: longitudinal effects). F: Study 3, cross-sectional effects. "*" indicates the significance level of the simple slope test (i.e., whether the simple slope is significantly different from zero). A: $^{*}ps < .001$. B: $^{*}p = .003$, $^{\#}p = .056$. C: $^{*}p < .001$, $^{\#}p = .056$. D: $^{*}ps < .029$. E. $ps > .077$. F. $^{*}ps < .001$.

References

Garcia, R. L., Kenny, D. A., & Ledermann, T. (2015). Moderation in the actor–partner interdependence model. *Personal Relationships, 22*(1), 8-29. doi:10.1111/pere.12060

Hooper, D., Coughlan, J., & Mullen, M. (2008). Structural equation modelling: Guidelines for determining model fit. *Articles*, 2.

Rosseel, Y. (2012). Lavaan: An R package for structural equation modeling and more. Version 0.5–12 (BETA). *Journal of Statistical Software, 48*(2), 1-36.
